# Asthma and atopy prevalence are not reduced among former tuberculosis patients compared with controls in Lima, Peru

**DOI:** 10.1186/s12890-019-0804-z

**Published:** 2019-02-13

**Authors:** Anthony L. Byrne, Ben J. Marais, Carole D. Mitnick, Frances L. Garden, Leonid Lecca, Carmen Contreras, Yaninna Yauri, Fanny Garcia, Guy B. Marks

**Affiliations:** 1 The University of Sydney, St. Vincent’s Hospital Sydney, Heart Lung Clinic, Xavier Building. 390, Victoria Street, 2010 Australia; 2 Socios En Salud Sucursal Perú, Partners In Health, Lima, Peru; 3 Centre for Research Excellence in Tuberculosis (TB-CRE), Sydney, Australia; 4 Blacktown Hospital Clinical School, University of Western Sydney, Sydney, Australia; 5 Marie Bashir Institute for Infectious Diseases and Biosecurity (MBI), University of Sydney, Sydney, Australia; 6 Harvard Medical School, Boston, MA USA; 7 The Woolcock Institute of Medical Research, Sydney, Australia; 8 South Western Sydney Clinical School, University of New South Wales, Sydney, Australia; 9 Ingham Institute of Applied Medical Research, Sydney, Australia; 10 Ministry of Health, Red de Salud Lima Ciudad, Lima, Peru

**Keywords:** Tuberculosis, Atopy, Allergy, Immune response, Asthma, Peru

## Abstract

**Background:**

Although there are theoretical reasons for believing that asthma and atopy may be negatively correlated with tuberculosis, epidemiological studies have had conflicting findings.

**Objective:**

To determine if people with confirmed tuberculosis were less likely to be atopic and less likely to have atopic disease including asthma compared to those with no previous tuberculosis.

**Methods:**

Patients in Lima, Peru with a prior history of tuberculosis were identified from clinic records in this cohort study. A representative sample of individuals without a prior tuberculosis diagnosis was recruited from the same community. Allergen skin prick testing was performed to classify atopic status. Allergic rhinitis was identified by history. Asthma was defined by symptoms and spirometry. Eosinophilic airway inflammation was measured using exhaled nitric oxide levels.

**Results:**

We evaluated 177 patients with, and 161 individuals without, previous tuberculosis. There was a lower prevalence of atopy among people with prior tuberculosis on univariate analysis (odds ratio 0.57; 95% confidence interval 0.37–0.88) but, after adjustment for potential confounders, this was no longer statistically significant (aOR 0.64, 95% CI 0.41–1.01). The prevalence of allergic rhinitis (aOR 0.76, 95% CI 0.47 to 1.24 and asthma (aOR 1.18, 95% CI 0.69 to 2.00) did not differ significantly between the two groups. We also found no significant difference in the prevalence of elevated exhaled nitric oxide (aOR 1.30, 95% CI 0.78 to 2.17) or a combined index of atopic disease (aOR 0.86, 95% CI 0.54 to 1.36).

**Conclusion:**

In this urban environment in a middle-income country, prior tuberculosis may be associated with a reduced risk of atopy but does not protect against asthma and atopic disease.

## Introduction

Atopy is epidemiologically associated with asthma, rhinitis and eczema [1–3]. It is estimated that asthma affects 334 million people worldwide and is one of the top 10 causes of death amongst young adolescents [4]. Allergic rhinitis is also common and often associated with asthma [5]. Some have hypothesized that a reduction in the prevalence of common bacterial infections (including tuberculosis), generally improved sanitation and/or a “westernized” lifestyle may underlie the increased frequency with which atopy and atopic disease are diagnosed [6, 7]. The “*hygiene hypothesis*” suggests that exposure to bacterial infections and toxins, which was common at the turn of last century and is still present in many developing countries today, provides an “infectious milieu” that reduces the development of atopy and atopic disease [8].

Tuberculosis is a common infectious disease that disproportionately affects people in developing countries [9, 10]. Whilst tuberculosis remains the biggest infectious disease killer on the planet, being responsible for an estimated 1.6 million deaths in 2017, the immunological effects of *M. tuberculosis* infection may influence the risk of developing atopy and atopic disease [11]. Tuberculosis is associated with a T-helper 1 (T_H_1) lymphocyte immune response dominated by cytokines such as interferon-γ [12]. There is evidence from observational studies of an inverse association between tuberculosis infection, as measured by a positive tuberculin skin test, and the presence of atopy, defined as measured an elevated serum IgE and/or a T_H_2 cytokine profile [13]. Furthermore, animal models using *M. vaccae* vaccination demonstrated that mycobacterial infection may inhibit features of allergic asthma [14]. Subsequent population-based studies have demonstrated a variable protective effect from mycobacterial exposure, either *Bacille Calmette-Guérin* (BCG) vaccination or *M. tuberculosis* infection, with the most consistent protective effect observed in those with a genetic pre-disposition to atopic disease [15–17].

Evidence of the effect of tuberculosis disease (as opposed to infection) on the risk of atopy or atopic disease is limited. Ecological studies have suggested a reduced risk of atopy and symptoms of asthma among populations with higher tuberculosis notification rates [18]. However, this has not been confirmed in studies of individuals with and without tuberculosis. In this study we determine the rate of atopy among a cohort of patients with laboratory confirmed tuberculosis using the gold standard of skin prick testing. The measurement of exhaled nitric oxide (FeNO) may provide some additional insight into the pathophysiology, as nitric oxide (NO) is produced in the respiratory tract by activated inflammatory cells such as alveolar macrophages and is known to be elevated in asthma. Interestingly, NO has also been found to have important anti-mycobacterial effects in murine models [19]. Human studies suggest that patients with active pulmonary TB have lower FeNO levels early in their disease [20], which perhaps represents a deficient respiratory inflammatory response, but further data is needed. The objective of this study was to determine whether individuals with a confirmed history of tuberculosis have a reduced risk of atopy and of atopic diseases compared with people without a history of tuberculosis.

## Methods

### Study design and setting

**Fig. 1:**
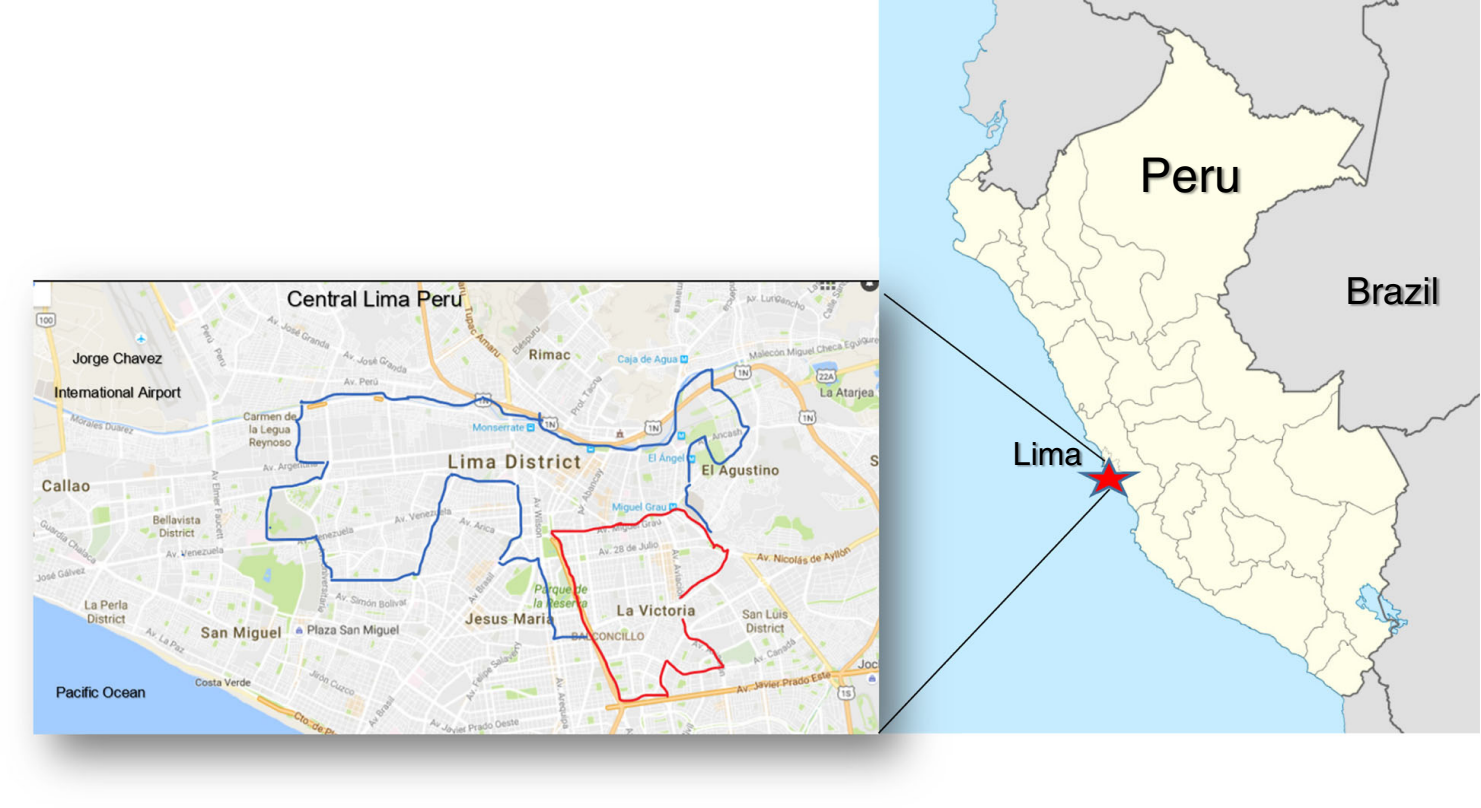
Study setting in central Lima, Peru. Lima District (blue); La Victoria (red)

We performed a population based, cross-sectional study in *La Victoria* and *Cercado de Lima,* two densely populated communities in central Lima (sea level; latitude 12.0^0^ S) with a combined area of around 50 km^2^ and a population of 500,000 (Fig. 1). The Pulmones Post Tuberculosis (PPTB) study (Protocol OEE-040-14) was approved by the Peruvian National Institute of Health Institutional Committee for Ethics in Investigations (Comité Institucional de Ética en Investigación, Instituto Nacional de Salud) and Partners In Health (Socios En Salud Sucursal, Peru).

### Study population

The study population included persons aged between 10 and 70 years who were residents of *La Victoria* or *Cercado de Lima*. Former tuberculosis patients in the communities were identified in from ministry of health records of public health centres (*Centros de Salud*) and comprised a random sample of patients with bacteriologically confirmed tuberculosis who had completed treatment for drug susceptible tuberculosis (DS-TB) in the 12 months prior to November 1st, 2014 and all patients who had completed treatment for multi-drug resistant tuberculosis (MDR-TB) in the 36 months before the 1st November 2014. Individuals without a history of tuberculosis were randomly selected from the general population of La Victoria and Cercado de Lima using a modified version of the “random walk” [21] sampling technique (see Appendix for a more detailed description).

### Atopy

We identified the presence of *atopy* by performing allergen skin prick tests [22, 23]. A single drop of (each) of 11 aeroallergen extracts (*Dermatophagoides pteronyssinus, Dermatophagoides farinae, Blomia tropicalis, Blatella germanica, Periplaneta americana, cat, dog, Grass mix 6, Lolium perenne, Aspergillus fumigatus, Alternaria* alternate (*Inmunotek* Pty Ltd) [24], one positive control (histamine) and one negative control (saline buffer/50% glycerol) was placed on the skin of volar surface of the participant’s forearm. Single use, disposable blood lancets were used to pierce the skin with an epidermis “prick and lift” technique at an angle of 45 degrees (*GF Health Products*, Inc) [25]. The reaction was read using a standardized measuring device after 15 min. Wheal size was quantified as the mean of the longest diameter and its perpendicular. Wheals that were ≥ 3 mm bigger than the negative control were considered positive. A negative test was recorded if there were no allergen wheals ≥3 mm bigger than the negative control and the positive control wheal was ≥3 mm bigger than the negative control wheal. If the positive control weal was < 3 mm bigger than the negative control wheal and there were no positive allergen wheals, the test was considered invalid. Participants with one or more positive wheals to allergen were considered to have atopy.

### Atopic disease

#### Allergic rhinitis

Participants were asked, "Do you usually have a blocked or runny nose, itchy eyes or sneezing when you DO NOT have a cold (in other words “hay fever” or allergic rhinitis) [26] and “Has a doctor or other health care provider ever told you that you have allergic rhinitis or hay fever?”. Participants who answered “Yes” to either of these questions were defined as having allergic rhinitis.

#### Asthma

Participants completed a questionnaire and had spirometry measured, before and 15 min after the administration of salbutamol 400mcg via metered dose inhaler. Spirometry was performed using an Easy-One spirometer (NDD™, Andover, United States of America) in accordance with American Thoracic Society (ATS) and European Respiratory Society (ERS) guidelines [27]. Only spirometric records that met ATS/ERS standards for quality and reproducibility (classified as scores A, B or C) were included in the analysis. All spirometric examinations were carried out with the subject seated and using a single-use mouthpiece (spirette). In addition, the fractional concentration of expired nitric oxide (FeNO) was measured using an Aerocrine device (NIOX-MINO™) per the manufacturer’s instructions in parts per billion.

The presence of asthma was defined as one or more of the following: a) a positive response to the question “In the last 12 months have you had whistling, blowing or wheeze in your chest?” (previous wheeze) [27]; b) a positive response to the question “Has a doctor or other health care provider ever told you that you have asthma, asthmatic bronchitis or allergic bronchitis?” (asthma diagnosis); or c) an increase in FEV_1_ > 200 ml and > 12% of baseline (compared to the pre-bronchodilator FEV_1_). High FeNO was defined as FeNO > 35 ppb for children under 18 years or > 50 ppb for adults. Intermediate FeNO was defined as ≥20 and ≤ 35 for children and ≥ 25 and ≤ 50 for adults. Values below 20 and 25, respectively, were defined as normal.

### Statistical methods

**Fig. 2:**
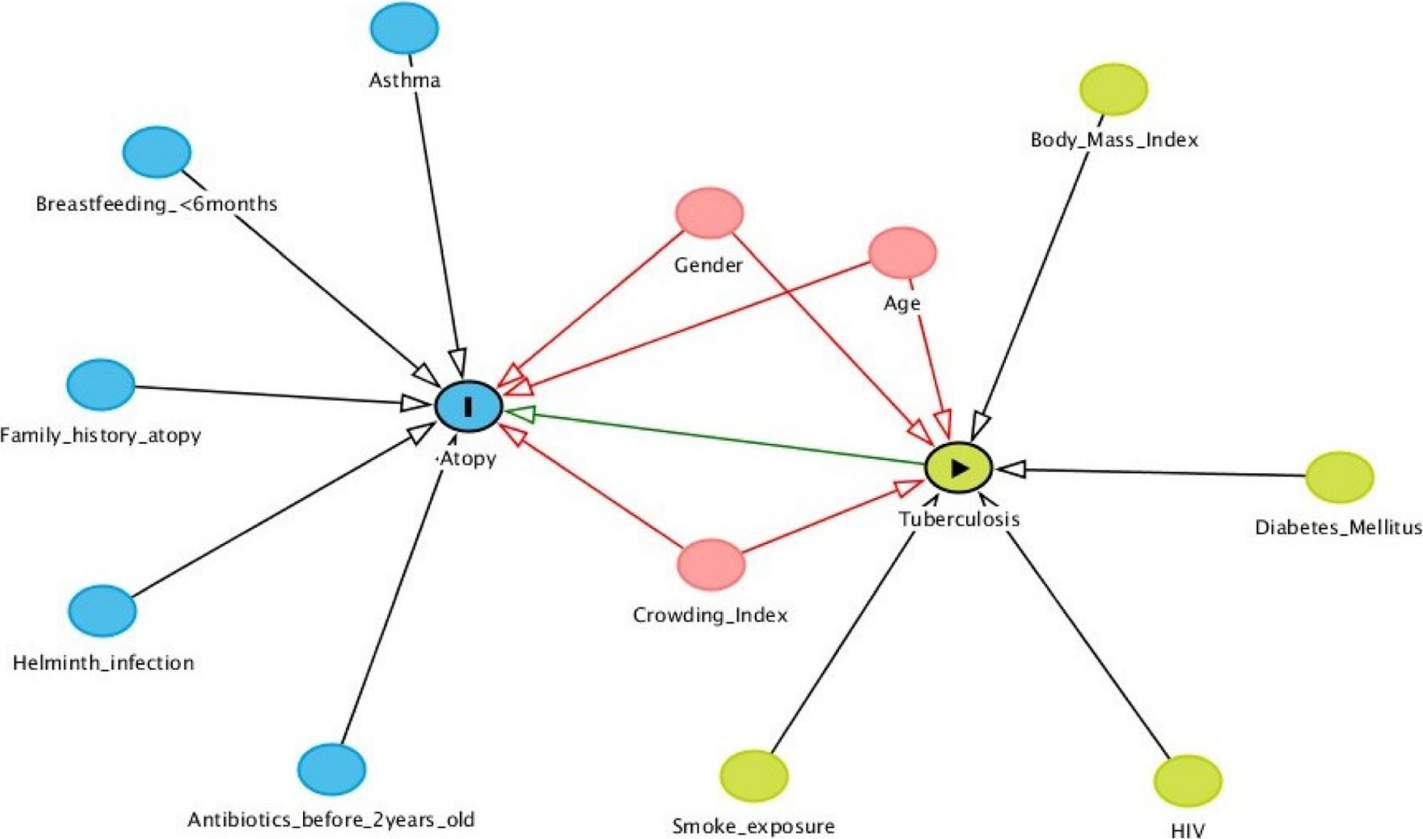
Causal directed acyclic graph linking tuberculosis (the exposure) to atopy (the outcome).  exposure (Tuberculosis),  outcome (Atopy),  ancestor of exposure,  ancestor of outcome,  ancestor of exposure *and* outcome,  adjusted variable, causal path,  unobserved (latent),  biasing path. Adjustment for confounders (ancestors of both the exposure and the outcome). Age, Crowding Index, Gender. These 3 co-variates were identified as the minimal sufficient adjustment sets required for estimating the total effect of Atopy on Tuberculosis. These co-variates (age, crowding index and gender) were therefore adjusted for in the multi-variate analysis

We constructed a directed acyclic graph (DAG) [28]. to guide the selection of covariates for multivariate analysis (Fig. 2). All data was analyzed using SAS version 9.4 (SAS Institute, Cary, NC) [29]. Multivariate logistic regression was implemented to estimate odds ratio (ORs) and 95% Confidence Intervals (95% CI). Linear regression was used to compare continuous outcomes. A combined index of atopic disease was calculated using the combination of both asthma and allergic rhinitis outcomes for each group. A *p* value of ≤0.05 was considered significant.

## Results

A description of enrolment of participants with prior tuberculosis has been published previously [30]. For the group without tuberculosis, we randomly selected 296 residents of the study area and 161 (54%) agreed to participate in the study. In total, the enrolled study population included 177 patients with a recent history of treated, laboratory-confirmed tuberculosis and 161 individuals without prior tuberculosis selected from the general population. The characteristics of the study population are described in Table 1. Those with prior tuberculosis tended to be younger (median age 29.0 compared to 37.6 years), included a higher proportion of males (57.6% vs 30.4%) and were more likely to be university educated (14.7% vs 3.7%) compared to those without prior tuberculosis. Participants in both groups were predominantly of mixed race (“mestizo”). The body mass index (BMI) was similar between groups as were the reported exposures to cigarette smoke, indoor air pollution and occupational dust.

**Table 1:** Demographic and baseline characteristics of participants with and without previous tuberculosis

Characteristic	No TB *N* = 161 *n* (%)^a^	TB *N* = 177 *n* (%)^a^	*p*-value*
Demographics			
Age median years (IQR) Children < 18 years	37.6 (20.7–52.5) 30 (18.6)	29.0 (20.6–43.0) 20 (11.3)	0.01
Sex (male)	49 (30.4)	102 (57.6)	< 0.01
Height; mean cm (SD)	158.1 (8.8)	161.1 (9.8)	NS
BMI; mean kg/m^2^ (SD)	26.0 (5.1)	24.5 (4.1)	NS
Born in Lima	112 (69.6)	130 (73.5)	NS
Race			
White	16/152 (10.5)	10/164 (6.1)	NS
Black	5/152 (3.3)	2/164 (1.2)	NS
Mestizo (mixed)	131/152 (86.2)	152/164 (92.7)	NS
Socioeconomic indicators			
Employed	75 (46.9)	117 (66.5)	< 0.01
Education			
Primary or less	31 (19.3)	16 (9.0)	NS
Secondary	89 (55.3)	96 (54.2)	NS
Technical	35 (21.7)	39 (22.0)	NS
University	6 (3.7)	26 (14.7)	< 0.01
Crowding Index median persons/m^2^ (IQR)	0.13 (0.08–0.23)	0.13 (0.08–0.25)	NS
Exposures			
Current smoker^b^	17 (10.6)	13 (7.4)	NS
Ever smoked	18 (11.3)	28 (16.0)	NS
Passive smoke	38 (23.8)	16 (9.0)	< 0.01
Indoor air pollution^c^	39 (24.7)	41 (23.4)	NS
Occupational dust^d^	40 (25.0)	59 (33.5)	NS

*TB* – tuberculosis, *BMI* – Body Mass Index, *SD*- Standard Deviation, - *IQR*- Inter Quartile Range

*Using the Chi^2^ test, NS – Not Significant (Chi^2^ > 0.05)

^a^unless stated otherwise; ^b^*s*moked ≥ 1 cigarette in the last 30 days; ^c^Indoor use of bio-combustible fuel such as wood-fire cooking-ever; ^d^ever worked in a dusty job

### Association of atopy or atopic disease (including asthma) with prior tuberculosis

On univariate analysis, the prevalence of atopy was lower among those with prior tuberculosis (76; 42.9%) than among those without a history of tuberculosis (90; 57.0%; *p* = 0.01). The difference in prevalence of specific antigen sensitization was only statistically significant for *Periplaneta americana* (7.9% vs 15.3%, *p* = 0.03). The prevalence of symptoms of allergic rhinitis (hay fever) was lower in participants with prior tuberculosis than in those without a history. of tuberculosis (23.3% vs 35.6%; *p* = 0.01), but there was no difference in the prevalence of doctor diagnosed allergic rhinitis. People with a history of prior tuberculosis were more likely to have a family history of allergic rhinitis or eczema than those without a history of tuberculosis (23.0% vs 12.1%; *p* = 0.01). Reported wheeze was common in this study population, but did not differ between those with and without prior tuberculosis. Similarly, the prevalence of a doctor diagnosed asthma, a positive bronchodilator response on spirometry, and elevated FeNO did not differ between participants with and without prior tuberculosis (Table 2).

**Table 2:** Prevalence of atopy and atopic disease by tuberculosis status

	No TB N = 161 n/N (%)	TB N = 177 n/N (%)	*p*-value*
Atopic disease			
Rhinitis symptoms	57/160 (35.6)	41/176 (23.3)	0.01
Rhinitis diagnosis	18/160 (11.3)	21/176 (11.9)	0.85
Previous wheeze	36/160 (22.5)	36/176 (20.5)	0.65
Asthma diagnosis	12/160 (7.5)	10/176 (5.7)	0.50
Positive bronchodilator response	5/139 (3.6)	7/159 (4.4)	0.72
Family history rhinitis/eczema	17/141 (12.1)	35/152 (23.0)	0.01
Atopy (positive skin prick tests, SPT)			
Positive SPT (any)	90/158 (57.0)	76 (42.9)	0.01
House dust mite			
Dermatophagoides farinae	43/157 (27.4)	47 (26.6)	0.86
Dermatophagoides pteronyssinus	49/157 (31.2)	50 (28.3)	0.55
Blomia tropicalis	36/156 (23.1)	39 (22.0)	0.82
Cockroach			
Blattella germanica	27/157 (17.2)	32 (18.1)	0.83
Periplaneta americana	24/157 (15.3)	14 (7.9)	0.03
Pets			
Cat dander	9/157 (5.7)	10 (5.7)	0.97
Dog dander	9/156 (5.8)	11 (6.2)	0.86
Grasses			
Ryegrass	4/158 (2.5)	3 (1.7)	0.59
Grass mix	7/157 (4.5)	5 (2.8)	0.42
Mould			
Aspergillus fumigatus	8/158 (5.1)	7 (4.0)	0.62
Alternaria alternata	10/156 (6.4)	6 (3.4)	0.20
Airway inflammation			
FeNO (ppb)^a^			
Normal	122/158 (77.2)	134/176 (76.1)	0.23
Intermediate	18/158 (11.4)	29/176 (16.5)	
High	18/158 (11.4)	13/176 (7.4)	

* Using the Chi^2^ test; ^a^Fraction of exhaled nitric oxide (ppb); normal (adults < 25; children < 20), intermediate (adults ≥25 and ≤ 50; children ≥20 and ≤ 35), high (adults > 50; children > 35)

In the DAG we found that gender, age and crowding index were potential antecedents of both tuberculosis and atopy, hence we included these variables as potential confounders in the multivariate model (Fig. 2). After adjustment for these potential confounders, the negative association between a history of tuberculosis and the presence of atopy was no longer statistically significant (adjusted odds ratio 0.64, 95% CI 0.41–1.01) (Table 3). For allergic rhinitis defined by either symptoms or a prior medical diagnosis there was also an attenuation of the effect following adjustment for age, gender and crowding index with neither univariate or multivariate analysis reaching statistical significance (adjusted odds ratio 0.76 95% CI 0.47–1.24). Similarly, there was no significant difference in the prevalence of asthma among former tuberculosis patients compared to community controls (adjusted odds ratio 1.18, 95% CI 0.69–2.00). Also, the prevalence of an elevated FeNO and the prevalence of a combined index of any atopic disease did not differ significantly between the two groups.

**Table 3:** Uni- and multi-variate logistic regression analyses assessing the association between atopy or atopic disease and tuberculosis status

Signs/symptoms of atopy	Tuberculosis^a^	
	Odds ratio (95% CI)	*p*-value
Atopy^b^		
Univariate^c^	0.57 (0.37, 0.88)	0.01
Multivariate^d^	0.64 (0.41, 1.01)	0.054
Allergic rhinitis^e^		
Univariate^c^	0.64 (0.41, 1.02)	0.06
Multivariate^d^	0.76 (0.47, 1.24)	0.28
Asthma^f^		
Univariate^c^	0.96 (0.59, 1.57)	0.87
Multivariate^d^	1.18 (0.69, 2.00)	0.55
Elevated FeNO^g^		
Univariate^c^	1.50 (0.94, 2.40)	0.09
Multivariate^d^	1.30 (0.78, 2.17)	0.32
Any atopic disease^h^		
Univariate^c^	0.70 (0.46, 1.08)	0.11
Multivariate^d^	0.86 (0.54, 1.36)	0.52

*CI* -confidence interval, *FeNO* – fraction of exhaled nitric oxide

^a^Included patients successfully treated for drug susceptible and drug-resistant disease; the cohort without tuberculosis (community controls) served as the reference group;

^b^A positive skin prick test (> 3 mm greater than the negative control) to any one of the following allergens; house dust mite (*D. farinae, D. pteronyssinus, B. tropicalis*), cockroach (*B. germanica, P. americana*), cat dander, dog dander, grasses (ryegrass, grass mix), or mould (*A. fumigatus, A. alternata*);

^c^Unadjusted univariate analyses;

^d^Multivariate analysis adjusted for crowding index, gender and age in accordance with the DAG in (Fig. 1);

^e^Symptoms of allergic rhinitis (hayfever) or prior medical diagnosis;

^f^Asthma symptoms of wheeze in the last 12 months or diagnosed asthma or a positive bronchodilator response recorded on spirometry;

^g^Elevated FeNO > 50 ppb (parts per billion) for adults or > 35 ppb for children (under 18 years); marker of airway inflammation;

^h^Either allergic rhinitis ^(e)^ or Asthma ^(f)^

## Discussion

We found a suggestion that people with prior tuberculosis had less atopy, as indicated by a positive skin prick test, than those with no history of tuberculosis. However, we did not find evidence of a decreased prevalence of any diseases associated with atopy, that is, allergic rhinitis and various manifestations of asthma, among people with recent tuberculosis.

Atopy is an IgE mediated immediate hypersensitivity response that occurs following exposure to environmental allergens [31]. Although others have observed that tuberculosis is associated with reduced manifestations of atopic disease, most previous studies have focused on the the consequences of latent tuberculosis infection rather than active tuberculosis [32, 33]. In contrast to previous studies, this study focused on patients with recent, laboratory confirmed, tuberculosis. The human immunological response to tuberculosis is complex, with important distinctions between latent infection and active disease [34]. People with active tuberculosis have increased numbers of circulating regulatory CD4 T cells (that express CD25 + FoxP3+) and a greater IFN-γ response, compared to those with latent infection [35, 36].

Peru is a middle-income country of South America that has high rates of asthma and allergic rhinitis despite routine BCG vaccination at birth [37]. Whilst the BCG vaccine is administered to reduce the risk of tuberculosis (particularly extrapulmonary disease among infants), limited evidence suggest that it may also reduce the risk of allergic rhinitis [17]. The average ambient temperature in Lima ranges between 16 and 32 °C year-round with high humidity, conditions optimal for mite reproduction [38]. The Peru Urban to Rural Asthma (PURA) study recently found that there were higher rates of asthma (12% versus 3%) and allergic rhinitis among 13–15-year-old children living in (urban) Lima than in a regional town on the north coast (Tumbes) [39]. In this context, it is noteworthy that both tuberculosis and house dust mites, may benefit from the environmental milieu found in the urban slums of Lima, Peru. The impact of tuberculosis disease on the risk of having asthma and related disorders has not been explored in epidemiological studies, other than in ecological analyses. Although the International Study of Asthma and Allergies in Childhood (ISAAC) [40] found that the prevalence of asthma in children was inversely related to the incidence of tuberculosis in 55 countries [41]. Our findings suggest that this question merits further study [42, 43].

We did not find any significant association between prior tuberculosis and any asthma-related outcomes, including wheezing, bronchodilator responsiveness, or exhaled nitric oxide (FeNO) concentration. It has been observed that people with prior tuberculosis may be more likely to have airflow obstruction [30]. Our FeNO findings imply that this is not mediated by eosinophilic inflammation. Any potential protective effect of tuberculosis on “atopic asthma”, may be lost due to lung damage and respiratory sequelae mediated through neutrophilic inflammation and other mechanisms [44]. This is counter to the idea of a reduced burden of atopic disease in settings where infectious diseases are common (the “hygiene hypothesis”). In fact, our findings suggest quite high rates of both atopy and atopic disease in this middle-income country. It would therefore not appear correct to ignore the possibility of atopic disease in patients with a history of tuberculosis. As this group has a greater risk of airflow obstruction and an equal risk of atopic disease (compared to community controls), respiratory health among former tuberculosis patients appears to be important. This may be an unmet need, especially in settings where the routine follow-up of tuberculosis patients does not occur or where linkage to (respiratory) specialist care is difficult.

As this was a cross-sectional study, we cannot comment on the temporal association between tuberculosis exposure and atopy or atopic disease. Our findings may also have been influenced by non-responder bias due to the relatively low participation rates. As only successfully treated tuberculoisis patients were recruited it is possible that patients with an unfavourable treatment outcome may have had different responses, but this has limited biological plausibility. We meticulously assessed and corrected other common factors that may have introduced bias, such as smoking and occupational dust exposure. Other study strengths include the recruitment of a well defined study population with controls subjects from the same community. All patients had laboratory confirmed tuberculosis, eliminating the risk of recall bias regarding the exposure. The population-based random sampling of controls participants from the same communities provided robust internal controls to assess the impact of tuberculosis on atopy and atopic outcomes, independent of other environmental, genetic and lifestyle factors. Another strength was the comprehensive assessment of atopy and atopic disease with standardized questionnaires and objective testing.

## Conclusions

The apparent dissociation between atopy and atopic disease, in their relationship to tuberculosis, emphasizes the importance of understanding the biological mechanisms that underpin this possible inverse association. Further work is required to elucidate these mechanisms.

## Abbreviations

95% CI: 95% Confidence Intervals
ATS: American Thoracic Society
BCG: *Bacille Calmette-Guérin*
DAG: Directed acyclic graph
DS-TB: Drug susceptible tuberculosis
ERS: European Respiratory Society
FeNO: Fraction of exhaled nitric oxide
FEV_1_: Forced Expiratory Volume in 1 s
ISAAC: International Study of Asthma and Allergies in Childhood
MDR-TB: Multi drug resistant tuberculosis
NO: Nitric oxide
OR: Odds Ratio
PPTB: Pulmones Post Tuberculosis (lungs after tuberculosis)
PURA: Peru Urban to Rural Asthma
